# Health professionals’ stigmatizing attitudes towards people with mental illness: A cross-sectional study in a referral hospital in Uganda

**DOI:** 10.1371/journal.pone.0313153

**Published:** 2024-12-02

**Authors:** Joan Abaatyo, Novatus Nyemara, Scholastic Ashaba

**Affiliations:** 1 Department of Psychiatry, Mbarara University of Science and Technology, Mbarara, Uganda; 2 Department of Psychiatry, King Ceasor University, Kampala, Uganda; Kwame Nkrumah University of Science and Technology, GHANA

## Abstract

**Background:**

Health professionals in primary care settings show stigmatizing attitudes towards people with mental illness (PMI), leading to undermined quality of care delivered. However, information is sparse on stigmatizing attitudes of health professionals towards PMI in Uganda. This study aimed to discover the levels of stigmatizing attitudes towards PMI and associated factors among health professionals in Uganda.

**Method:**

We enrolled 254 health professionals at Mbarara Regional Referral Hospital in a cross-sectional study. Community attitude towards mental illness-2 (CAMI-2) scale was used to assess stigmatizing for attitudes. Linear regression was used to determine factors associated with level of stigmatizing attitudes.

**Results:**

The average overall CAMI score for all participants was 91.1±16.6. Nurses/midwives compared to doctors had significantly higher total CAMI score (p<0.001), and higher malevolent (p = 0.01) and non-acceptance attitudes (p = 0.02) than doctors. Doctors had significantly lower authoritarian attitudes than clinical officers, (p = 0.004). Being male (aCoef: -4.86; p = 0.02), increase in compassion satisfaction (aCoef: -0.44; p = 0.02), and increased mental health knowledge (aCoef: -2.90; p = <0.001), increased likelihood of having lower levels of stigmatizing attitudes, while being a non-psychiatric health professional was associated with higher levels of stigmatizing attitudes (aCoef: 12.08; p = 0.01).

**Conclusion:**

Health professionals exhibit moderate levels of stigmatizing attitudes towards PMI and stigmatizing attitudes are more among nurses/midwives. Various steps including providing education and training on mental illness, promoting community integration and social inclusion, and advocating for policies, should be taken to reduce stigmatizing attitudes of health professionals towards PMI.

## 1. Introduction

Worldwide, health professionals in primary care settings show stigmatizing attitudes toward people with mental illness (PMI) [1, 2]. Manifestations of some of these stigmatizing attitudes among health professionals have been reported, extending from absolute denial to mental health care, provision of poor-quality medical care, and physical and verbal abuse, to making some PMI wait longer for services or passing their care over to junior colleagues [3]. Additional stigmatizing attitudes towards PMI by health care professionals include exclusion from decisions, coercive treatment with subtle or overt threats, giving insufficient information about any other condition or treatment options, treating these patients in a paternalistic or demeaning manner, and telling them they would never get well [4]. Stigmatizing attitudes against PMI among health professionals can differ based on the causes of these attitudes, how they are expressed, and the effects they have [5–7]. Drivers of stigmatizing attitudes towards PMI include fear, negative beliefs, lack of knowledge about the mental health conditions, inability to clinically manage the conditions that the PMI have, and lack of awareness of the effects of their stigmatizing attitudes towards PMI [8–10]. Health professionals’ stigmatizing attitudes can also be facilitated by moral concerns derived from their personal disagreement of behaviors, for example; PMI have been considered by health professionals to be unpredictable, threatening, without restraint, and violent [11, 12]. Limited availability of the training required to offer care to PMI in general medical undergraduate courses and medical training also causes many of the health professionals to devalue, dismiss and dehumanize PMI with whom they come in contact [13].

Consequently, the stigmatizing attitudes of health professionals towards PMI leads to social disqualification of these patients and has direct negative consequences on the treatment, recovery, rehabilitation, social reintegration, and impaired quality of life [14, 15]. The more PMI feels stigmatized, the lower their self-regard and confidence, social re-integration and quality of life and this negatively affects the help-seeking behaviors due to fear of being considered weak or the fear of discrimination [14, 16]. Despite the detrimental effects of these stigmatizing attitudes, many health professionals exhibit them and are uncomfortable dealing with PMI [1]. As a result, the health professionals’ capabilities for constructive health-care provision are affected, thereby compromising the quality of care they offer to the PMI [17, 18].

Despite the fact that mental health treatments are offered in regional referral hospitals in Uganda [19], the majority of general health care practitioners do not view these services as urgent. For example, during the COVID-19 pandemic MOH officials (majority being health professionals) transformed most psychiatric units at regional referral hospitals into COVID-19 isolation centers [20]. Information on stigmatizing attitudes of health professionals towards PMI in Uganda is scarce despite the abundance of evidence of these attitudes among health professionals in low-and-middle income countries [1, 14, 21]. Therefore, this study aimed to determine the health care professionals’ stigmatizing attitudes towards PMI and its associated factors in Uganda.

## 2. Methods

### 2.1 Study design and setting

This was a cross-sectional study conducted at Mbarara Regional Referral Hospital (MRRH) in southwestern Uganda, from 21^st^ November, 2022 to 28^th^ February, 2023. MRRH is the biggest public hospital in Mbarara located 270km away from Kampala, the capital city of Uganda. The hospital serves multiple districts in western Uganda and the neighboring countries of Burundi, Rwanda, Democratic Republic of Congo (DRC), and Tanzania.

### 2.2 Study population and eligibility screening

We recruited health professionals (specifically doctors, nurses/midwives, clinical officers, interns, and residents) offering health services at MRRH at the time of study. We excluded undergraduate medical and nursing students, health professionals who were sick, and those who were on work leave at the time of the study.

### 2.3 Sample size

The sample size was determined using Kish Leslie formula [22] for finite populations. Assuming a type, I error of 5%, a significance level at p<0.05, an absolute error or precision of 5%, and a 25% non-response rate the minimum sample size required to replicate this analysis was 254 participants.

### 2.4 Sampling procedure and data collection

Potential participants were contacted either in their office or during unit/clinical meetings. The selection of participants was based on the participants’ willingness to participate. Prior to completing the questionnaire, all participants were required to provide consent. The questionnaire was self-administered and on average, took approximately 30 minutes to complete. Before collecting data, the questionnaire was pretested with ten health professionals who were not part of the final sample. Based on their feedback, minor adjustments were made to the wording of some questions to improve clarity without altering their intended meaning. Data was collected by the the principal investigator assisted by a trained research assistant. To avoid any interference with patient care or work obligations, the health professionals were interviewed at the time most convenient to them most especially during lunch break or at the end of their scheduled duties.

### 2.5 Study variables

The questionnaire was developed for this study and it included different sections including the sociodemographic details (age, gender, marital status, specific health profession, and work experience), clinical factors (years of clinical work experience, personal and family history of mental illness, past experience working in a mental health unit, further training in psychiatry, WHO Mental-Health gap (MHGAP) training, mental health knowledge, personal experience with mental illness (living with or being diagnosed with a mental health condition), weeks of psychiatry training attained in school), and community attitudes towards mental illness [23].

### 2.6. Study measures

#### 2.6.1 Community Attitude to Mental Illness (CAMI-2)

A previously validated and widely utilized questionnaire, the CAMI-2, was used to assess for health care professionals’ attitudes towards patients with mental illness [24]. The CAMI was originally developed for use with the general population but has been used with various samples of health professionals [25]. The CAMI-2 questionnaire is made up of 40 statements, each rated on a 5-point Likert-scale that ranges from “1 = Strongly Agree” to “5 = Strongly Disagree". It aims to provide systematic insights into community attitudes towards patients with mental illness by measuring four factors (subscales): 1) Authoritarianism (AU), 2) Benevolence (BE), 3) Social Restrictiveness (SR), and 4) Community Mental Health Ideology (CMHI). The questionnaire contains 10 items from each factor that distinguish between respondents with "stigmatizing" and "non-stigmatizing" attitudes towards patients with mental illness. Each factor has five statements expressing pro- and five expressing anti-sentiments. Reverse scoring is applied to the anti-sentiment statements for the dimensions of AU and SR, and for the pro sentiments on the dimensions of BE and CMHI. AU refers to the view of mentally ill people as inferior and requiring coercive handling, BE refers to a paternalistic and sympathetic view of the mentally ill, SR is the belief that the mentally ill are a threat to society and should be avoided, and CMHI refers to acceptance of mental health services and mentally ill patients in the community, including the impact of mental health facilities in residential areas. Higher mean scores on each dimension suggest stigmatizing attitudes, and higher overall scores indicate more stigmatizing attitudes towards patients with mental illness. Higher BE scores are associated with a less sympathetic (malevolent) and humanistic view of PMI and higher CMHI scores are associated with non-acceptance of mental health services and PMI in the community.This questionnaire was preferred for this study because of its ability to capture the complexity and multidimensionality of stigmatizing attitudes and because it has been rated as having moderate to good internal consistency, with α values of 0.68 for AU, 0.88 for CMHI, 0.80 for SR, and 0.76 for BE [26]. In this study, the scale showed very good internal consistence with a Cronbach’s alpha of 0.85. The subscales for authoritarianism, benevolence, social restrictiveness and community mental health ideology had Cronbach’s alpha values of 0.42, 0.64, 0.77 and 0.81, respectively.

#### 2.6.2 Mental Health Knowledge Questionnaire (MHKQ)

The MHKQ was used to screen for mental health knowledge of the health professionals. It was developed to evaluate public knowledge and awareness of mental health by the Chinese Ministry of Health (MOH) in 2009 [27]. It contains 16 self-administered items which require participants to select “true,” “false,” or “unknown” about statements concerning mental health. Higher scores indicated higher MHK. The Cronbach’s alpha of MHKQ was reported to be 0.61[28] and in this study it was 0.52. Higher scores indicate higher mental health knowledge.

#### 2.6.3 Professional Quality of Life Scale-5 (ProQOL-5)

To assess the quality of life of medical workers, the Professional Quality of Life Scale-5 (ProQOL-5) was utilized. It is the most often used indicator of the benefits and drawbacks of working with individuals who have gone through incredibly trying circumstances. The ProQOL, which is intended for people in helping or caring professions, consists of 30 items measuring various aspects of compassion satisfaction and compassion fatigue [29]. Three subscales of the ProQOL assess compassion burnout, compassion satisfaction, and compassion fatigue. The findings of the scales cannot be merged to produce a single meaningful score because each subscale is distinct. The test consists of 30 items, with 10 on each scale, and is scored numerically on a Likert scale with a range of 1 (never) to 5 (very often). This study found that respondents who scored higher on the Compassion Fatigue subscale had a higher probability of developing compassion fatigue. Higher scores on the burnout subscale indicated that the individual was at risk of experiencing burnout symptoms (e.g., hopelessness, helplessness) [30]. Higher scores on the compassion satisfaction subscale indicate the respondent was experiencing better satisfaction with his or her ability to provide care. Alpha reliabilities for the scale structure reported are as follows; for the Burnout (a  =  0.75), Compassion Fatigue (a  =  0.81), and Compassion Satisfaction (a  =  0.88) [29]. In this study, the Cronbach’s alpha values of burnout, compassion satisfaction and compassion fatigue were 0.60, 0.79 and 0.74 respectively.

### 2.7 Ethical considerations

The study was conducted in accordance with the Declaration of Helsinki 2013 [31]. The study received ethical approval from Mbarara University of Science and Technology Research Ethics Committee/ (MUST-REC) under approval number #MUST-2022-555, before the study began. The hospital director granted permission to collect data from participants before the study began. All participants provided voluntary written informed consent before enrolment into the study.

### 2.8 Data analysis

Data from the questionnaires were coded appropriately into the Microsoft 2016 Excel spreadsheet and later transferred into the Stata version 15 for statistical analysis. Numerical data were summarized using means and standard deviations for normally distributed continuous data or medians and interquartile ranges for continuous skewed variables. Nominal data were summarized using proportions. The Kruskal-Wallis’s test was performed to compare medians while analysis of variance (ANOVA) was performed to compare means. Based on the highest score expected for the total CAMI score, less than the 25th percentile (i.e., less than 50) was considered as low levels, 25^th^ to75^th^ percentile (50–150) was considered as moderate and more than 75th percentile (more than 150) was considered as high levels stigmatizing attitudes. To ascertain which specific categories of medical professionals differ from one another regarding stigmatizing sentiments, Turkeys post hoc analysis was conducted. Means based on the total CAMI score were obtained to determine level of stigmatizing attitudes. Before running the multiple linear regression models, the relationships between all continuous and dichotomous independent variables were examined using the Spearman rank correlation coefficient test to check for collinearity. High Spearman correlations were found between the following variables: further training in psychiatry and WHO Mental health gap (MHGAP) training (r = 0.85). Due to the multicollinearity in the data, MHGAP training was removed from the multiple linear regression models. All hypothesis testing was conducted assuming a <0.05 significance level.

## 3. Results

The distribution of health professionals was as follows: doctors (52.0%), nurses/midwives (42.9%), and clinical officers (5.1%). The mean age of the participants was 32.7±7.7) years. There were more male health professionals in this survey (52.8%) and majority were doctors, and the median duration of clinical experience among these health professionals was 7.4±6.5 years. Doctors had more weeks of training in psychiatry during school than nurses/midwives and clinical officers (median of 4 vs 3 vs 3). Doctors had more Mental health knowledge than nurses/midwives and clinical officers (mean of 14 vs 13.2 vs 3.2) **(Table 1).**

**Table 1:** Participant characteristics distribution across the health profession.

Variable	All participants = 254	Health profession		
		Doctors	Nurses/ midwives	Clinical officers
	n (%)			
		132 (52.0%)	109 (42.9%)	13 (5.1%)
**Age** (*Mean*, *SD*)	32.8 (7.7)	31.7 (5.9)	33.8 (9.2)	34.3 (9.4)
**Gender**				
Female	120 (47.2)	34 (28.3)	81 (67.5)	5 (4.1)
Male	134 (52.8)	98 (73.1)	28 (20.9)	8 (5.9)
**Marital status**				
Not married	116 (45.7)	66 (56.9)	43 (37.1)	7 (6.0)
Married/living with partner	138 (54.3)	66 (47.8)	66 (47.8)	6 (4.3)
***Clinical factors***				
Years of clinical work experience (*Median*, *IQR*)	5 (3–10)	5 (3–6)	7 (3–12)	5 (2–12)
Weeks of training in psychiatry (*Median*, *IQR*)	3.2 (2–4)	4 (4–5)	3 (2–3)	3 (2–3)
Mental health knowledge (*Mean*, *SD*)	13.7 (1.5)	14 (1.2)	13.2 (1.7)	13.5 (1.1)
Had further training in psychiatry				
No	202 (79.5)	109 (54.0)	83 (41.1)	10 (4.9)
Yes	52 (20.5)	23 (44.2)	26 (50.0)	3 (5.8)
Had MHGAP training				
No	200 (78.7)	110 (55.0)	80 (40.0)	10 (5.0)
Yes	54 (21.3)	22 (40.7)	29 (53.7)	3 (5.6)
Health profession classification				
Psychiatry health professionals	18 (7.1)	10 (55.6)	5 (27.8)	3 (16.7)
Non-psychiatry health professionals	236 (92.9)	122 (51.7)	104 (44.1)	10 (4.2)
Experience working in a psychiatric unit				
No	207 (81.5)	113 (54.6)	86 (42.5)	8 (3.9)
Yes	47 (18.5)	19 (40.4)	23 (49.0)	5 (10.6)
Positive family history of Mental illness				
No	194 (76.4)	106 (54.6)	78 (40.2)	10 (5.1)
Yes	60 (23.6)	26 (43.3)	31 (51.7)	3 (5.0)
Personal experience with mental illness *(living with or being diagnosed with a mental health condition)*				
No	198 (78.3)	110 (55.6)	79 (39.9)	9 (4.5)
Yes	55 (21.7)	21 (38.2)	30 (54.5)	4 (7.3)
***Professional quality of life***				
Compassion Satisfaction (*mean*, *SD*)	39 (5.5)	39 (4.8)	40 (6.2)	42 (5.0)
Compassion fatigue (*mean*, *SD*)	23 (6.7)	23 (6.8)	24 (6.5)	23 (7.35)
Burnout (*mean*, *SD*)	26 (4.62)	26 (4.5)	27 (4.5)	24 (5.8)

*SD* = Standard deviation, IQR = Interquartile range

### 3.1 Health professionals’ attitude toward PMI

The average CAMI score for all participants was 91.1±16.6; which lies between the 25^th^ and 75^th^ percentile. The highest and lowest mean scores for all participants were measured on authoritarianism (26.8±5.0) and benevolence (19.0±4.9) respectively. This indicated that health professionals regarded the mentally-ill as being somewhat “inferior” requiring a “coercive” approach and they harbor more sympathetic views towards PMI. There was a significant difference among the means of the CAMI and the attitudes of AU, BE and CMHI. There was no statistically significant difference in the SR attitude among the health professions (**Table 2**).

**Table 2:** Stigmatizing attitudes across health professions.

Variables	Health profession				p-value
	All participants	Doctor	Nurse/midwife	Clinical officer	
Total CAMI score (*mean/SD*)	91.1 (16.6)	86 (16.8)	95.6 (15.2)	97.5 (15.5)	**<0.001**
***CAMI score subscales*** (mean/ SD)					
Authoritarianism	26.8 (5.0)	25.1 (4.7)	28.5 (4.5)	29.5 (5.6)	**<0.001**
Benevolence	19.0 (4.9)	18.1 (5.0)	20.0 (4.4)	21.0 (5.9)	**0.01**
Social restrictiveness	22.0 (6.0)	21.2 (6.1)	22.8 (5.5)	22.7 (4.6)	0.12
Community mental health ideology	23.3 (6.2)	22.2 (6.4)	24.4 (5.9)	24.2 (4.5)	**0.03**

Tukey’s posthoc test revealed a significant difference in the total CAMI score between Nurses/midwives and Doctors (p = <0.001, Mean difference = 8.9±2.1), a significant difference in AU attitude between Doctors and Clinical officers (p = 0.004, Mean difference = -4.3±1.3) and between nurses/midwives and Doctors (p = <0.001, Mean difference = 3.4±0.6).). There was also a significant difference in BE and CMHI attitude between nurses/midwives and Doctors i.e. (p = 0.01, Mean difference = 1.8±0.6) and (p = 0.02, Mean difference = 2.1±0.8) **(Table 3).**

**Table 3:** Comparison of stigmatizing attitudes among health professions.

Variable	Comparison					
	Doctor vs Clinical Officer		Nurse/midwife vs Clinical officer		Nurse/midwife vs Doctor	
	Mean dif. (S.E)	p-value	Mean dif. (S.E)	p-value	Mean dif. (S.E)	p-value
Total CAMI score	-10.7 (4.7)	0.06	-1.8 (4.7)	0.92	8.9 (2.1)	**<0.001**
Authoritarianism	-4.3 (1.3)	**0.004**	-0.9 (1.4)	0.77	3.4 (0.6)	**<0.001**
Benevolence	-3.0 (1.4)	0.09	-1.1 (1.4)	0.70	1.8 (0.6)	**0.01**
Community mental health ideology	-2.0 (1.8)	0.52	0.2 (1.8)	0.99	2.1 (0.8)	**0.02**

### 3.2 Factors associated with severity of overall stigmatizing attitudes towards PMI based on total CAMI scores

At multivariable analysis, being male, compassion satisfaction and increased mental health knowledge were significantly associated with having lower levels of stigmatizing attitudes (aCoef: -4.86; 95% CI: -8.82 –-0.89; *p* = 0.02), (aCoef: -0.44; 95% CI: -0.82 –-0.06; *p* = 0.02), and (aCoef: -2.90; 95% CI: -4.27 –-1.53; *p* = <0.001), respectively, while being a non-psychiatric health professional was associated with higher levels of stigmatizing attitudes (aCoef: 12.08; 95% CI: 2.60–21.55; *p* = 0.01) **(Table 4).**

**Table 4:** Linear regression analysis for factors associated with stigmatizing attitudes.

Variable	Bi variable analysis		Multivariable analysis	
	Coef. (95% CI)	p-value	Coef. (95% CI)	P-value
Age	-0.01 (-0.28–0.25)	0.93	-0.01(-0.45–0.42)	0.95
*Sex*				
Female	1 (reference)		1 (reference)	
Male	-5.98(-10.04–-1.92)	0.004	-4.86 (-8.82 –-0.89)	**0.02**
*Marital status*				
Not married	1 (reference)		1 (reference)	
Married/Living with partner	3.44 (-6.45–13.33)	0.49	1.92 (-2.19- –6.04)	0.36
Years of clinical work experience	0.03 (-0.30–0.35)	0.87	-0.55 (-0.59–0.48)	0.84
Duration of psychiatry training	-0.05 (-0.15–0.04)	0.25	0.06 (-0.04–0.16)	0.84
Mental health knowledge	-4.01 (-5.30 - -2.72))	<0.001	-2.90 (-4.27 –-1.53)	**<0.001**
*Had further training in psychiatry*				
No	1 (reference)		1 (reference)	
Yes	-4.94 (-10.01–0.13)	0.06	-2.56 (-7.99–2.87)	0.35
*Health profession classification*				
Psychiatric health professional	1 (reference)		1 (reference)	
Non-psychiatric health professional	14.28 (6.45–22.11)	<0.001	12.08 (2.60–21.55)	**0.01**
*Past experience working in a psychiatric unit*				
No	1 (reference)		1 (reference)	
Yes	-4.07 (-9.35–1.21)	0.13	1.86 (-4.19–7.09)	0.54
*Family history of mental illness*				
No	1 (reference)		1 (reference)	
Yes	-3.76 (-8.58–1.07)	0.13	-2.42 (-6.96–2.13)	0.30
*Personal experience with mental illness (living with or being diagnosed with a mental health condition)*				
No	1 (reference)		1 (reference)	
Yes	-2.23 (-7.24–2.77)	0.38	-3.18 (-7.77–1.41)	0.17
*Professional quality of life*				
Compassion satisfaction	-0.81 (-1.17 –-0.44)	<0.001	-0.44 (-0.82 - -0.06)	**0.02**
Compassion fatigue	0.68 (0.38–0.98)	<0.001	0.22 (-0.13–0.57)	0.22
Burnout	0.96 (0.53–1.39)	<0.001	0.40 (-0.10–0.90)	0.11

## 4 Discussion

This study examined the stigmatizing attitudes of health professionals towards PMI. The results indicate that doctors, nurses/midwives, and clinical officers hold stigmatizing attitudes towards PMI. Health professionals had more authoritarian and less malevolent attitudes towards PMI with nurses/midwives having more authoritarian, malevolent and non-acceptance attitudes towards PMI compared to doctors. However, in this study, there was no difference in social restrictiveness attitudes among the different health professionals. The study also found that being male, compassion satisfaction and higher mental health knowledge were associated with lower levels of stigmatizing attitudes towards PMI, while being a non-psychiatry health professional was associated with higher levels of stigmatizing attitudes towards PMI.

The average CAMI score of 91.1±16.6 among all health professionals suggests that, on average, the surveyed health professionals held moderate levels of stigmatizing attitudes towards mental illness. This echoes findings from previous studies, and could imply that there is room for improvement in reducing stigma within the healthcare community [32, 33]. The higher scores on authoritarianism could stem from societal misconceptions about mental illness, inadequate education, or inadequate exposure to PMI [34, 35]. This attitude might lead to a preference for restrictive interventions rather than therapeutic and supportive approaches [35]. The finding that health professionals harbor more sympathetic views towards PMI reflects a genuine concern for the well-being of PMI due to unequal treatment and resources allocation between mental and physical health care in Uganda [36].

The finding that compared to doctors, nurses/midwives had more authoritarian, malevolent and non-acceptance attitudes echoes the findings of a study in a teaching hospital in Greece by Arvaniti et al, 2008 [37], where nurses were found to have more stigmatizing attitudes towards PMI than doctors [37]. This is likely due to differences in education and training as doctors typically receive more training in psychiatry and mental health than nurses and midwives do [32]. Additionally, nurses and midwives spend more time with patients than doctors [33] increasing chances of having more direct contact with patients who exhibit symptoms of mental illness lead to a greater sense of frustration, fear, or stigmatization related to the behaviour that is often displayed by patients with mental illness [34]. Further more, nurses and midwives often work long hours, in high-stress environments with limited medical supplies, and may be under-resourced and overworked [35] which can lead to frustration, burnout and result in negative attitudes towards PMI. This study also found that clinical officers had more authoritarian attitudes than doctors. This is also likely attributable to differences in education and training as clinical officers in Uganda receive a diploma as their highest academic qualification and are hence unlikely to get exposure to mental health training due to limited duration of training [38].

The finding that there was no difference in social restrictiveness attitudes among the different health professionals is likely because of limited exposure to PMI professions as in this study the median duration of psychiatry training was less than 5 weeks. This also echoes findings in previous studies among non-psychiatric health professionals which depict that limited exposure to PMI causes health professionals to believe that people with mental illness are a threat to society and should be avoided [25, 34, 35].

The finding that higher mental health knowledge was associated with lower stigmatizing attitudes towards PMI is likely due to the fact that mental health knowledge reduces stigmatizing attitudes of health professionals towards PMI which echoes findings of previous research [39–41]. Overall mental health knowledge improves understanding of the nature of mental illness, building greater confidence in their ability to care for PMI through a more compassionate and informed lens, rather than through stigmatizing beliefs and stereotypes [42]. This could also explain the finding in this study that being a non-psychiatric health professional was associated with more stigmatizing attitudes towards PMI.

However, the finding that male gender was associated with lower stigmatizing attitudes was contrary to the findings in other studies [14, 43]. This is likely because the study had more men and many of these were doctors, hence had had more exposure to mental health information and people with mental illness. Furthermore, traditional gender roles may lead men to view mental illness differently than women. For example, men may be socialized to be more stoic and less emotional [44], which may make them less likely to stigmatize people with mental illness. Lastly, male health professionals in Uganda may be held to higher professional standards of behaviour and ethics [45], which could discourage stigmatizing attitudes towards PMI.

The finding that higher levels of compassion satisfaction was associated with lower stigmatizing attitudes echoes findings from previous studies [46], possibly because health professionals who experience high levels of compassion satisfaction have a greater sense of personal accomplishment in their work, a greater sense of pride in the care they provide and may develop a greater commitment to providing high-quality care to patients including PMI [47].

### 4.1 Strengths and limitations

The current research has certain limitations including: the study was cross-sectional; no causal link could be established between increased stigmatizing attitudes and other factors. Therefore, larger, more comprehensive prospective studies are recommended. Additionally, the sample of the health professionals may not necessarily represent the experiences and attitudes of health professionals in the country since the study participants were selected from a single health facility. However, the selected health facility serves as a teaching hospital and therefore may replicate views of various other health professionals to a certain level. One other potential research limitation is that despite obtaining a high Cronbach’s alpha for the total CAMI scale and other subscales, the authoritarian subscale within the measure demonstrates a low Cronbach’s alpha. Therefore, findings specifically related to authoritarianism may be less reliable compared to the results based on the other subscales or the total scale. Future refinement of the authoritarian subscale in this setting is recommended. In addition, the instruments used to assess the attitude, and knowledge about mental illness has not been validated for use in the Ugandan context. Although some parameters were found to be significant in the regression models, they only explain a small percentage of the constructs on the CAMI scale. As such, there may be other factors that were not measured which could influence the scores for CAMI factors, such as cultural influences[48–50]. The findings from this study highlight the need for greater awareness and education among healthcare professionals regarding the needs of people with mental illness and the role of stigmatization in the development and outcome of mental disorders.

## 5. Conclusion

In general, health professionals exhibit moderate levels of stigmatizing attitudes toward PMI, could imply that there is room for improvement in reducing stigma within the healthcare community. Stigmatizing attitudes were more among nurses and midwives as compared to other health professionals. By recognizing and addressing stigmatizing attitudes towards patients with mental illness, health professionals can provide better mental health care and support to all individuals, regardless of their mental health status. Various steps can be taken to reduce stigmatizing attitudes of health professionals towards PMI. These include providing education and training on mental illness, promoting community integration and social inclusion, and advocating for policies that reduce stigma and discrimination towards individuals with mental illness.

## Abbreviations

PMI: *People with mental illness*
CAMI: *Community Attitudes towards Mental illness*
AU: *Authoritarianism*
BE: *Benevolence*
SR: *Social restrictiveness*
CMHI: *Community Mental Health Ideology*
MRRH: *Mbarara Regional Referral Hospital*
